# Neurofibromatosis type 1 adult surveillance form for Austria

**DOI:** 10.1007/s00508-024-02443-0

**Published:** 2024-09-12

**Authors:** Vincent Sunder-Plassmann, Amedeo A. Azizi, Said Farschtschi, Robert Gruber, Markus Hutterer, Viktoria Ladurner, Claas Röhl, Tobias Welponer, Anna-Sophie Bergmeister-Berghoff

**Affiliations:** 1 Division of Oncology, Department of Medicine I, Medical University of Vienna, Waehringer Gürtel 18-20, 1090 Vienna, Austria; 2 Christian Doppler Laboratory for Personalized Immunotherapy, Department of Medicine I, Medical University of Vienna, Vienna, Austria; 3 Division of Neonatology, Pediatric Intensive Care and Neuropediatrics, Department of Pediatrics and Adolescent Medicine, Medical University of Vienna, Vienna, Austria; 4 Comprehensive Center for Pediatrics, Medical University of Vienna, Vienna, Austria; 5 Comprehensive Cancer Center, Medical University of Vienna, Vienna, Austria; 6 International Center for Neurofibromatoses, Department of Neurology, University Medical Center Hamburg-Eppendorf, Hamburg-Eppendorf, Germany; 7 Department of Dermatology, Venereology and Allergy, Medical University of Innsbruck, Innsbruck, Austria; 8 Department of Neurology with Acute Geriatrics, Saint John of God Hospital Linz, Linz, Austria; 9 Department of Neurology, Hospital of Villach, Villach, Austria; 10 NFKinder, Vienna, Austria; 11 Department of Dermatology and Allergology, University Hospital of the Paracelsus Medical University, Salzburg, Austria

**Keywords:** Neurofibromatosis type 1, Prevention, Screening, Cancer, Tumor predisposition syndromes

## Abstract

**Background:**

Neurofibromatosis type 1 (NF1) is a rare autosomal dominant tumor predisposition syndrome with a birth prevalence of approximately 1 in 2000–3000 individuals. Management of both benign and malignant tumors arising in individuals with NF1 is demanding and tumors may be difficult to treat. Both standardized and individual surveillance programs are therefore highly important to prevent morbidity and mortality in patients with NF1.

**Methods:**

The guidelines for the clinical management of NF1 recently proposed by the European Reference Network for Genetic Tumor Risk Syndromes provide the cornerstone of the present surveillance form and were discussed through three rounds of voting and a final consensus meeting involving experts from five Austrian and one German clinical NF1 centers for adults and one patient organization representative. Subsequently, 31 items within 4 categories were integrated into the proposed surveillance form for Austria. All recommendations, unless otherwise specified, pertain to primarily asymptomatic patients in routine follow-up.

**Recommendations:**

At healthcare transition from pediatric to adult surveillance or the initial visit in adulthood, we suggest a thorough clinical, laboratory and radiological examination to obtain a baseline for future diagnostics. To comply with the general screening recommendations in Austria, we suggest extending the frequency of clinical visits from annual to biennial at 50 years of age. In cases of clinical dynamics, early follow-up is recommended to facilitate early detection of potential complications. Particular emphasis should be placed on preventive patient education.

**Supplementary Information:**

The online version of this article (10.1007/s00508-024-02443-0) contains supplementary material, which is available to authorized users.

## Introduction

With approximately 1 in 2000–3000 individuals affected, neurofibromatosis type 1 (NF1) is one of the most common hereditary cancer syndromes [1]. Approximately 50% of patients inherit the disorder in an autosomal dominant way, while the remaining 50% of patients have de novo mutations of the *NF1* gene [2]. Even though NF1 is caused by a pathogenic variant in a single gene, the broad mutational spectrum of *NF1* transcripts results in a heterogeneous clinical phenotype of affected individuals, making the prediction of disease trajectory for the individual patient largely unpredictable [3]. Notably, with over 3000 known constitutional variants of the *NF1* transcript, only few are currently considered relevant predictors of health outcomes [4].

**Fig. 1:**
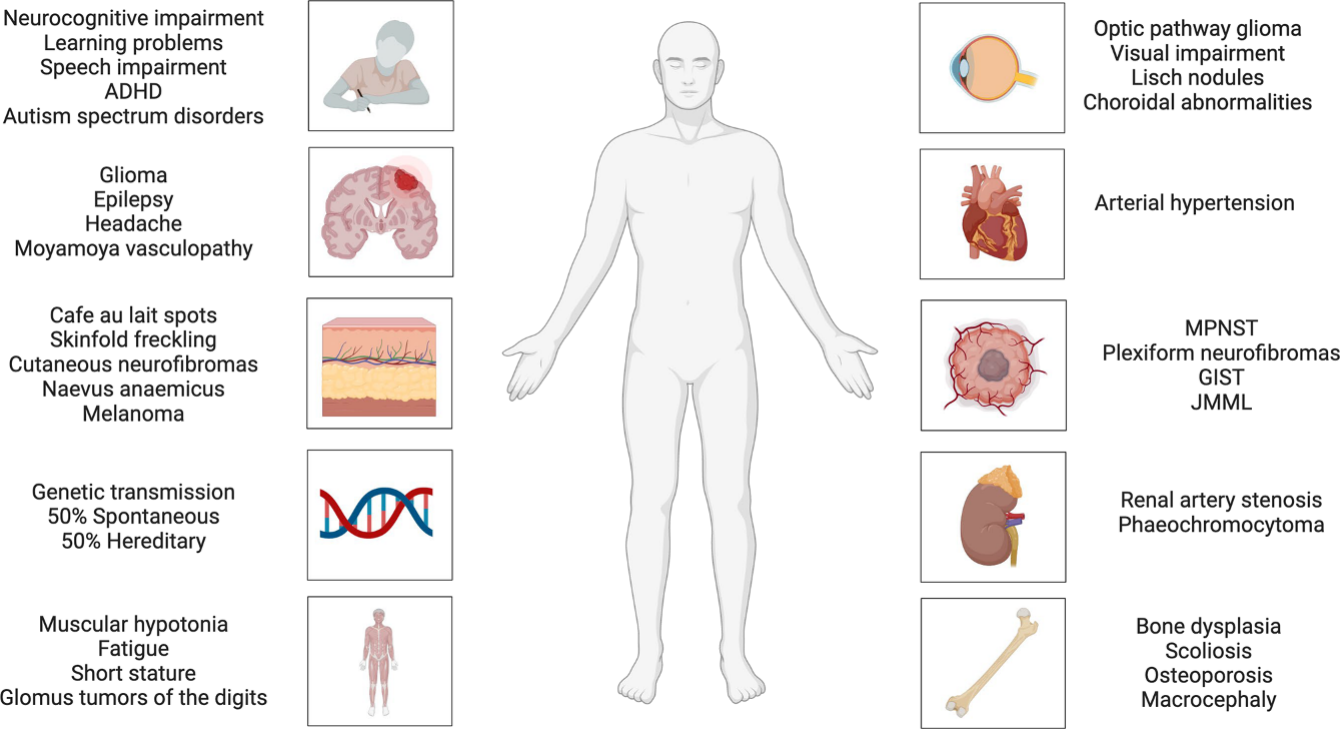
Manifestations of neurofibromatosis type 1. *ADHD* attention deficit hyperactivity disorder, *GIST* gastrointestinal stromal tumor, *JMML* juvenile myelomonocytic leukemia, *MPNST* malignant peripheral nerve sheath tumor

At present, NF1 can be diagnosed by the presence of ≥ 2 of the revised diagnostic criteria: ≥ 6 bilateral café au lait macules (CALM), bilateral skinfold freckling, (plexiform) neurofibromas, e.g. (sub-)cutaneous, nerve roots, plexus and/or peripheral nerves, optic pathway gliomas (OPGs), nodules or choroidal abnormalities, bone dysplasia, the presence of a heterozygous pathogenic *NF1* variant or the presence of parent with NF1 (Fig. 1; Table 1; [5]).

**Table 1:** Diagnostic criteria for neurofibromatosis type 1 in adults

**A** ≥ *2 of the following criteria (if no parent was diagnosed with NF1)*
**B** ≥ *1 of the following criteria if a parent is diagnosed with NF1*
≥ 6 Café au lait macules ≥ 15 mm in diameter in postpubertal individuals
Axillary or inguinal freckling^a^
≥ 2 Neurofibromas or ≥ 1 plexiform neurofibroma
Optic pathway glioma
≥ 2 Lisch nodules or choroidal abnormalities
≥ 1 Distinct osseous lesion (sphenoid dysplasia^b^, anterolateral bowing of the tibia, long bone pseudoarthrosis)
Heterozygous pathogenic NF1 variant with a VAF of 50% in apparently normal tissue such as white blood cells

^a^If only café au lait macules and freckling are present, with ≥ 1 of both being bilateral, NF1 is the most likely diagnosis. In some cases, the individual might have an alternative diagnosis such as Legius syndrome

^b^In case of ipsilateral orbital plexiform neurofibroma, sphenoid wing dysplasia is not a separate criterion. *NF1* neurofibromatosis type 1, *VAF* variant allele frequency. Adapted from Legius et al. [5]

The broad disease spectrum is not limited to these diagnostic criteria, but moreover includes neurocognitive impairments (learning disabilities, autism spectrum disorders, behavioral disorders, attention-deficit hyperactivity disorders), psychosocial stressors and, importantly, the (early) development of malignancies [6–8]. Notably, the predominant clinical features of scoliosis, cutaneous neurofibromas and large inoperable plexiform neurofibromas are accompanied by (neuropathic) pain and esthetic considerations, necessitating a multidisciplinary therapeutic concept including pharmacological, surgical and (neuro)psychological specialties.

It was recently shown that approximately one third of individuals with NF1 developed a non-neurofibroma neoplasm and 7.2% multiple non-neurofibroma neoplasms, at a younger age, compared to the general population. Neoplasms such as low-grade and high-grade gliomas, malignant peripheral nerve sheath tumors (MPNST), gastrointestinal stroma tumors (GIST), breast cancer or pheochromocytomas are long known to have a higher incidence in individuals with NF1 than in healthy control populations [8, 9].

The increased risk and in consequence morbidity of malignancies also accounts for the decreased average life expectancy by 10–15 years for affected patients [10]. The management of both benign and malignant tumors arising in individuals with NF1 is demanding and furthermore accompanied by burdensome neuropathic pain, deformities and psychosocial stress. The surgical management of complex nerve sheath tumors is often challenging, especially if diagnosed late, and radiotherapy carries a high risk of secondary malignancies for individuals with germline mutations. Systemic chemotherapy has also shown limited efficacy in several NF1-associated malignancies. Surveillance is therefore of utmost importance in the management of individuals with NF1 and is fundamental for the clinical management [11, 12].

Nevertheless, about two thirds do not develop malignancies and have a life expectancy comparable to otherwise healthy individuals. This cohort likely requires significantly fewer diagnostic efforts. To date, it is a major challenge to determine the appropriate level of comprehensive screening, clinical visits, diagnostics, and ultimately therapeutic interventions for each patient, to maximize tangible benefits and avoid unnecessary pathologization of individual predisposition.

Recent efforts to establish prognostic cohorts have correlated some specific genetic alterations, such as type 1 *NF1* deletions, to a more severe phenotype, but the prognosis of the majority of affected individuals is largely unknown [13]. Both standardized and individual screening approaches are therefore of importance to limit morbidity and mortality in NF1. Recently, national and international guidelines and recommendations for systematic monitoring of potentially affected organ systems have been developed that have significantly improved clinical care of patients with NF1 by standardizing and balancing screening procedures [14–16]. Although these resources provide comprehensive screening recommendations for individuals with NF1, they may lack applicability and have some shortcomings and variations when compared to each other.

The 2023 European Reference Network on Genetic Tumor Risk Syndromes (ERN GENTURIS) tumor surveillance guidelines for individuals with NF1 [15] provide a valuable, evidence-based decision-making resource for physicians treating patients with NF1. Based on this, our goal was to provide physicians treating adult individuals with NF1 in Austria a comprehensive, yet feasible surveillance form and guidance by integrating evidence-based national and international recommendations.

## Methods

### Objective and target population of the neurofibromatosis type 1 adult surveillance form

The objective of the neurofibromatosis type 1 adult surveillance form was to provide a comprehensive, easy to implement and evidence-based guidance for physicians treating individuals with NF1 in Austria. The recommendations are directed at adult individuals ≥ 18 years of age starting at healthcare transition (HCT) from pediatric care with an established diagnosis of NF1, without signs and symptoms suggestive of malignancy, or individuals ≥ 18 years of age with an established diagnosis of NF1 and no previous contact to a center of expertise. To account for the general Austrian healthcare screening recommendations, we have subclassified the target population into individuals below and above 50 years of age, where general screening for malignant diseases, particularly breast cancer screening, is expanded.

### Development of the screening form

**Fig. 2:**
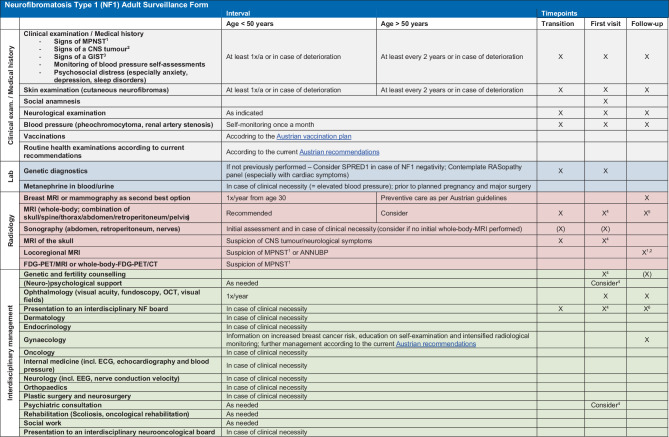
The Neurofibromatosis Type 1 Adult Surveillance Form for Austria ^1^ Signs suggestive of MPNST: rapidly growing or painful nodules, new neurological deficits, changes in consistency (e.g., new nodule in soft neurofibroma). ^2^ Signs suggestive of a CNS tumor: New focal neurological symptoms, de novo continuous headache, epileptic seizures, neurocognitive changes. ^3^ Signs suggestive of GIST: changes in digestion continuous abdominal pain, unintentional weight loss. ^4^ If not previously performed. ^5^ High-risk patient for MPNST development if 1 of the following criteria is met: previous atypical neurofibroma (ANNUBP); high internal tumor load; large or multiple plexiform neurofibromas; history of radiotherapy; a relative with NF1 and MPNST; NF1 microdeletion (including SUZ 12) or missense variant affecting codons 844–848. ^6^ In complex cases, unclear imaging, high-risk constellations. *ANNUBP* atypical neurofibromatous neoplasm of uncertain biological potential, *CNS* central nervous system, *CT* computed tomography, *EEG* electroencephalography, *EKG* electrocardiography, *FDG*-*PET* fluorodeoxyglucose positron emission tomography, *GIST* gastrointestinal stromal tumour, *MPNST* malignant peripheral nerve sheath tumor, *MRI* magnetic resonance imaging, *NF* neurofibromatosis, *OCT* optical coherence tomography

**Fig. 3:**
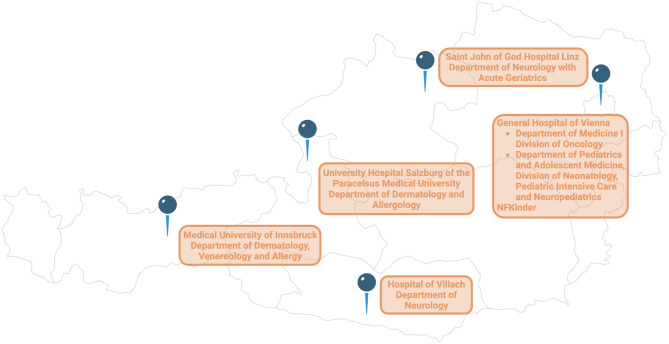
Austrian clinical centers and patient organization involved in the iterative consensus making process for the NF1 Adult Surveillance Form for Austria

The recently published guidelines by the ERN GENTURIS [15] provided the fundament of this surveillance form (Fig. 2; German version provided as Supplementary Fig. 1). In an iterative decision-making process, Austrian NF1 experts from five clinical centers and one patient organization (Fig. 3) discussed the relevance of each identified aspect in the context of the Austrian healthcare system. Following discussion with a German NF1 expert, 3 voting rounds and 1 consensus meeting, 4 categories (clinical examination and anamnesis; laboratory parameters; imaging; consultation of further medical specialties) with a total of 31 items pertinent to clinical care for NF1 patients were included in the final form.

## The neurofibromatosis type 1 adult surveillance form for Austria

### Healthcare transition

Healthcare transition (HCT) from child and family centered medicine to adult patient centered medicine constitutes a challenge and paradigm shift for individuals with NF1 [17].

While some complications arising in individuals with NF1 are associated with a younger age, others are more commonly encountered in adulthood (e.g., MPNSTs, cardiovascular problems, GISTs, breast cancer) [8]. Efforts aiming at the early detection of possible complications must thus change from those in adolescence. Yet, a comprehensive anamnesis and clinical examination including a neurological and skin examination remain the cornerstone in the early detection of complications in NF1 and must be considered at HCT and every subsequent clinical visit. Anamnesis and clinical examination at HCT should comply with our proposed approach described below. Furthermore, the initiation or completion of genetic testing should be offered to each patient. Monthly home blood pressure measurements should be assessed at and continued after HCT as a screening tool for pheochromocytoma and renal artery stenosis.

For the monitoring of plexiform neurofibromas, performing a whole-body (WB) or equivalent MRI at HCT is recommended to determine the internal tumor burden as a predictor for MPNST development. Similarly, cranial MRI is recommended at HCT to monitor glioma development. For patients presenting later in life, a baseline WB or spinal MRI may be considered and discussed individually, if no prior WB or spinal MRI surveillance was undertaken. If internal plexiform neurofibromas are absent, further examination by WB or spinal MRI is not necessary as the overall risk of MPNST is considered low. If a high internal tumor load is detected at HCT, refined screening programs utilizing WB-MRI, regional MRI, positron emission tomography (PET) MRI or PET/computed tomography (CT) should be considered if symptoms emerge or worsen. Importantly, the absence of high internal tumor burden on WB-MRI at transition does not exclude the formation of MPNST later in life [15, 18]. If whole body MRI or CT is not readily available, sonography might be considered as modality of choice [19]. In the case of developing a malignancy, discussion of each case in an interdisciplinary tumor board is strongly recommended.

Further emphasis should be placed on encouraging adults with NF1 to adhere to the recommendations of routine health check-ups and adhere to the recommended vaccination guidelines.

### Anamnesis and clinical examination

In accordance with published recommendations, we suggest annual clinical examinations for adults with NF1 ≤ 50 years and at least biennial assessment for individuals above the age of 50 years, including a focused neurological and dermatological examination. Each assessment should address the present and longitudinal development of clinical manifestations and symptoms, and their effects on quality of life.

Cutaneous neurofibromas are benign tumors originating from Schwann cell significantly affecting patient quality of life that are commonly cited as the leading psychological stressors for NF1 patients. A dermatological examination by inspection and palpation of the skin should therefore be performed at every visit. Laser surgery or further therapeutic options should be discussed with every patient if burdensome.

Similarly, a thorough neurological examination including a pain anamnesis must be considered at every assessment. The detection of focal neurological symptoms (e.g.. muscle weakness or paralysis, poor coordination, loss of sensation, pain, confusion or altered levels of consciousness) per clinical neurological examination is both cost-efficient and elegant before initiating further diagnostics. Importantly, (neuropathic) pain secondary to plexiform neurofibroma progression, scoliosis or neuropsychiatric or psychosocial conditions should be addressed early and treated accordingly. Similarly, the screening for, diagnosis and treatment of neuropsychiatric disorders such as depression, anxiety or sleep disorders, both primary and secondary to chronic (neuropathic) pain, should be actively assessed.

Not all tumors related to NF1 are commonly encountered in adult patients. Therefore, we suggest focusing on clinical signs and symptoms associated with the transformation of plexiform neurofibromas to MPNSTs (increase in size or change of consistency of pre-existing neurofibromas, pain, new neurological symptoms).

Yet, not only the malignant transformation, but also the continuous progression of benign plexiform neurofibromas should be monitored, and pharmacological and surgical treatment initiated when deemed clinically necessary. Additionally, we suggest screening for the development of CNS tumors (focal neurological symptoms, novel/changing headaches, epileptic seizures, neurocognitive changes) and GISTs (changes in digestion or stool consistency, abdominalgia, weight loss) [20] before initiating imaging, as well as glomus tumors of the digits (pain, localized tenderness, sensitivity to cold) [15]. Importantly, as 1–5% of individuals with NF1 develop pheochromocytoma during their lifetime [21] and NF1 predisposes to the development of renal artery stenosis, we suggest monthly monitoring of blood pressure at home, which should be checked at every clinical visit to enable early detection of arterial hypertension.

### Laboratory examination

Currently, the diagnostic work-up when suspecting NF1 usually includes comprehensive genetic testing and *NF1* transcript analysis. If the genetic work-up has not been adequately completed, we recommend completion according to local SOPs at HCT or first contact. If Legius syndrome is suspected (≥ 6 bilateral CALMs with or without skinfold freckling without further manifestations suggestive of NF 1), sprouty related EVH1 domain containing 1 *(SPRED1)* sequencing to confirm or exclude Legius syndrome is recommended. If cardiac anomalies are evident, thorough examination via the available RASopathy panel is suggested.

If pheochromocytoma is suspected based on blood pressure measurements or imaging, we recommend measuring blood or urine metanephrine levels. Of particular note, measurement of catecholamine levels should also be considered and discussed before planned pregnancy and/or surgery. Routine laboratory examinations at clinical visits are not recommended if the individual is asymptomatic.

### Imaging modalities

The NF1 inherently increases the risk of developing malignancies. It is therefore important to limit diagnostic radiation exposure whenever possible [22]. Therefore, MRI should be preferred over CT scans, especially in young patients.

Annual WB-MRI is not recommended for every individual; however, it might be indicated in selected high-risk constellations, such as confirmed atypical neurofibromatous neoplasm with uncertain biological potential (ANNUBP) [23], a high internal tumor burden detected by WB-MRI at HCT or high-risk genetic constellations, such as *NF1* microdeletions that affect the entire *NF1* gene and several flanking genes or a full deletion of the *NF1* gene [24]. Furthermore, if there is clinical suspicion of malignant transformation of symptomatic neurofibromas to MPNST, it is strongly recommended to perform regional or PET MRI. Of note, ultrasound guided assessment provides a cost-efficient and radiation free alternative if MRI is not readily available.

For women, annual breast cancer screening should be initiated at 30 years of age, which differs from the recommendations for breast cancer screening in the general population. As the presence of neurofibromas generally limits interpretability of traditional mammography, it is recommended to use MRI with contrast for breast cancer screening instead of mammography [25]. As the risk for breast cancer of the general and the NF1 population converges with increasing age, breast imaging above the age of 50 years should be performed according to the same guidelines as for the general population [26].

### Multidisciplinary management

Of particular note, NF1 is a very heterogeneous condition requiring a multidisciplinary approach to optimize clinical management. Hence, consultation of further medical specialties is strongly advised:

While there are currently no licensed pharmacological therapeutics available to alleviate the burden of cutaneous neurofibromas, various dermatologic and surgical procedures, such as laser ablation, surgical removal, electrodessication or radiofrequency ablation are well established for the treatment of cutaneous neurofibromas. Consultation with dermatologists and plastic surgeons therefore are a cornerstone to provide optimal care to patients with NF1.

Approximately one in five individuals with NF1 develops an OPG at a young age [27]. In adulthood, intracranial tumors such as OPGs or pilocytic astrocytomas typically undergo negligible alterations compared to those observed in pediatric NF1 patients or non-NF1 patients. As recommended by the Austrian Chamber of Physicians for individuals at risk of ophthalmologic disease, we recommend a clinical examination by a trained ophthalmologist at least once a year, in line with screening recommendations for pediatric patients with OPGs. If no OPG is evident at baseline cMRI, ophthalmologic examination should be initiated if clinical symptoms become apparent. Examinations should include testing for best corrected visual acuity, a fundus examination, perimetry, and optical coherence tomography, if available. Performing cMRI is recommended if abnormalities are detected to screen for (optic) glioma [28], with subsequent neurosurgical consultation, if deemed necessary.

Pheochromocytomas, among various other complications requiring an interdisciplinary management, present a significant challenge. While clinical detection is straightforward using routine blood pressure measurements, catecholamine levels and imaging, emerging endocrinological dysbalances should be evaluated and managed in agreement with an endocrinologist.

Aside from organic manifestations, the psychosocial burden associated with NF1 needs to be addressed [29]. Therefore, it is recommended to provide neuropsychological, psychiatric, and social services to each patient if deemed necessary or requested. As NF1 is inherited in an autosomal dominant manner [30], the transmission risk to the offspring should be addressed, and genetic counselling and fertility clinic consultations (including counselling regarding birth control) should be offered to affected individuals. To provide individuals with NF1 with an out-of-hospital point of contact, we furthermore strongly encourage clinicians and patients to connect with patient organizations. Patient organizations, such as NFKinder (www.nfkinder.at) in Austria, provide patients with valuable information, enable exchange with others affected and support patients to get access to specialized healthcare and treatment.

## Practical implications

The striking heterogeneity, ranging from asymptomatic disease course over decades, to burdensome and sometimes life-threatening complications at a young age necessitates targeted surveillance efforts for children, adolescents [31] and adults with NF1. Indeed, early detection of malignancies has proven to be the most valuable resource to mitigate morbidity and mortality in NF1 to date [16]. Yet, it is crucial to carefully balance the need to provide meaningful diagnostics for some patients with NF1, while less extensive diagnostic efforts might be sufficient for other patients with NF1. Therefore, systematic surveillance efforts must be supported by strong evidence, and the potential impact on patients and the healthcare system should be weighed against the benefits. The recently published ERN GENTURIS tumor surveillance guidelines for individuals with NF1 [15] provide compelling evidence for the proposed screening efforts.

## Supplementary Information

**Supplementary Fig. 1: German version of the Neurofibromatosis Type 1 Adult Surveillance Form for Austria ***1* *Zeichen für MPNST: schnell wachsende oder schmerzende Knoten, neue neurologische Ausfälle, Veränderung der Konsistenz (z.* *B. neuer Knoten in weichem Neurofibrom). 2* *Zeichen für einen ZNS Tumor: neue fokale Symptomatik, neue, starke kontinuierliche Kopfschmerzen, epileptischer Anfall, neuro-kognitive Veränderungen. 3* *Hinweis auf GIST:Veränderung in der Verdauung, kontinuierliche Bauchschmerzen, ungewollter Gewichtsverlust. 4* *Falls vorher keine Anbindung an ein Expertise NF1 Zentrum. 5* *Hochrisikopatient für MPNST: 1 Kriterium erfüllt: vorhergegangenes atypisches Neurofibrom (ANNUBP) od. hohe interne Tumorlast bzw. große oder multiple plexiforme Neurofibrome od. st.p. Strahlentherapie od. ein Verwandter mit NF1 und MPNST od. NF1-Mikrodeletion (incl. SUZ12) od. Missense Variante betreffend Codons 844–848. 6* *Bei komplexen Fällen, unklarer Bildgebung, high risk Konstellation. ANNUBP* atypical neurofibromatous neoplasm of uncertain biological potential;* CT* computed tomography*; EEG* electroencephalography*; EKG electrocardiography; FDG-PET* fluorodeoxyglucose positron emission tomography,* GF* visual field,* GIST gastrointestinal stromal tumor, MPNST* malignant peripheral nerve sheath tumor,* MRT* magnetic resonance imaging,* NF* neurofibromatosis,* OCT* optical coherence tomography,* RR* blood pressure,* WS* spinal,* ZNS* central nervous system.
