# Supplementary material for: Neurofibromatosis type 1 adult surveillance form for Austria
Source: Wien Klin Wochenschr. 2024 Sep 12;137(15-16):487–94. doi: 10.1007/s00508-024-02443-0 (PMC12370825; doi:10.1007/s00508-024-02443-0)
Supplement: Supplementary file 1 — Supplementary Fig. 1: German version of the Neurofibromatosis Type 1 Adult Surveillance Form for Austria 1 Zeichen für MPNST: schnell wachsende oder schmerzende Knoten, neue neurologische Ausfälle, Veränderung der Konsistenz (z. B. neuer Knoten in weichem Neurofibrom). 2 Zeichen für einen ZNS Tumor: neue fokale Symptomatik, neue, starke kontinuierliche Kopfschmerzen, epileptischer Anfall, neuro-kognitive Veränderungen. 3 Hinweis auf GIST:Veränderung in der Verdauung, kontinuierliche Bauchschmerzen, ungewollter Gewichtsverlust. 4 Falls vorher keine Anbindung an ein Expertise NF1 Zentrum. 5 Hochrisikopatient für MPNST: 1 Kriterium erfüllt: vorhergegangenes atypisches Neurofibrom (ANNUBP) od. hohe interne Tumorlast bzw. große oder multiple plexiforme Neurofibrome od. st.p. Strahlentherapie od. ein Verwandter mit NF1 und MPNST od. NF1-Mikrodeletion (incl. SUZ12) od. Missense Variante betreffend Codons 844–848. 6 Bei komplexen Fällen, unklarer Bildgebung, high risk Konstellation. ANNUBP atypical neurofibromatous neoplasm of uncertain biological potential; CT computed tomography; EEG electroencephalography; EKG electrocardiography; FDG-PET fluorodeoxyglucose positron emission tomography, GF visual field, GIST gastrointestinal stromal tumor, MPNST malignant peripheral nerve sheath tumor, MRT magnetic resonance imaging, NF neurofibromatosis, OCT optical coherence tomography, RR blood pressure, WS spinal, ZNS central nervous system. [file 508_2024_2443_MOESM1_ESM.docx]

| **Neurofibromatose Typ 1 (NF1) Erwachsenen Vorsorgebogen** | | | | | | | |
| --- | --- | --- | --- | --- | --- | --- | --- |
|  | | **Intervall** |  | **Zeitpunkte** | | | |
|  |  | **Alter < 50 Jahre** | **Alter > 50 Jahre** | **Transition** | **Erstvorst.** | **Kontrolle** |  |
| **Klinik/Anamnese** | **Klinische Kontrolle / Anamnese**   - Hinweis auf MPNST^1^ - Hinweis auf ZNS Tumor^2^ - Hinweis auf GIST^3^ - Kontrolle der Blutdruck-Selbstkontrollen - Psychosoziale Belastung (insbesondere Angst, Depression, Schlafstörung) | mind. 1x/a bzw. bei Verschlechterung | mind. Alle 2 Jahre bzw. bei Verschlechterung | X | X | X |  |
|  | **Hautstaus (Kutane Neurofibrome)** | mind. 1x/a bzw. bei Verschlechterung | mind. Alle 2 Jahre bzw. bei Verschlechterung | X | X | X |  |
|  | **Sozialanamnese** |  | |  | X |  |  |
|  | **Neurostatus** | Bei Bedarf | | X | X | X |  |
|  | **Blutdruck (Phäochromozytom, Nierenarterienstenose)** | Selbstkontrolle einmal pro Monat | | X | X | X |  |
|  | **Impfungen** | Lt. [Österreichischem Impfplan](https://www.sozialministerium.at/Themen/Gesundheit/Impfen/Impfplan-%C3%96sterreich.html) | |  |  |  |  |
|  | **Gesundenuntersuchung laut aktueller Empfehlung** | Lt. [Österreichischer Empfehlung](https://www.gesundheit.gv.at/leben/gesundheitsvorsorge/vorsorgeuntersuchung/was-wird-gemacht.html#untersuchungsprogramm) | |  |  |  |  |
| **Labor** | **Genetik Diagnostik** | Falls bisher nicht erfolgt – SPRED1 falls NF1 neg; RASopathie Panel erwägen (insb. bei kard. Sympt.) | | X | X |  |  |
|  | **Metanephrine im Blut/Harn** | bei klin. Notwendigkeit (= RR Erhöhung), bei geplanter Schwangerschaft; vor großer OP diskutieren | |  |  |  |  |
| **Radiologie** | **MRT Mamma (1. Wahl) oder Mammographie (2. Wahl)** | 1x/a ab 30 LJ | Vorsorge laut Österreichischer Empfehlung |  |  | X |  |
|  | **MRT (Ganzkörper; Kombination Schädel/ ges. WS/ Thorax/ Abdomen/ Retroperitoneum/ Becken)** | empfohlen | erwägen | X | X^4^ | X^5^ |  |
|  | **Sonographie (Abdomen, Retroperitoneum, ev. Nerven)** | Ausgangsbefund, dann bei klin. Notwendigkeit (Erwägen falls initial kein Ganzkörper MRT erfolgt) | | (X) | (X) |  |  |
|  | **MRT Schädel** | V.a. ZNS Tumor/ neurologischer Symptomatik | | X | X^4^ |  |  |
|  | **MRT (lokal)** | V.a. MPNST^1^ oder ANNUBP | |  |  | X^1,2^ |  |
|  | **FDG-PET/MR oder Ganzkörper-FDG-PET/CT** | V.a. MPNST^1^ | |  |  |  |  |
| **Interdisziplinäres Management** | **Humangenetische Beratung und Kinderwunsch Beratung** |  | |  | X^4^ | (X) |  |
|  | **(Neuro)Psychologische Betreuung** | Bei Bedarf | |  | Erwägen^4^ |  |  |
|  | **Ophtalmologie (Visus quant., Fundi, OCT, GF)** | 1x/a | |  | X | X |  |
|  | **Vorstellung interdisziplinäres NF Board** | bei klin. Notwendigkeit | | X | X^4^ | X^6^ |  |
|  | **Dermatologie** | bei klin. Notwendigkeit | |  |  |  |  |
|  | **Endokrinologie** | bei klin. Notwendigkeit | |  |  |  |  |
|  | **Gynäkologie** | Information erhöhtes Mamma Ca Risiko, Edukation Selbstabtastung; sowie bei klin. Notwendigkeit, Frequnz gemäß [Österreichischer Empfehlung](https://www.gesundheit.gv.at/leben/gesundheitsvorsorge/vorsorgeuntersuchung/was-wird-gemacht.html#untersuchungsprogramm) | |  |  | X |  |
|  | **Hämatologie-Onkologie** | bei klin. Notwendigkeit | |  |  |  |  |
|  | **Innere Medizin (inkl. EKG/Herzecho und RR Abklärung)** | bei klin. Notwendigkeit | |  |  |  |  |
|  | **Neurologie (inkl. EEG, Nervenleitgeschwindigkeit)** | bei klin. Notwendigkeit | |  |  |  |  |
|  | **Orthopädie** | bei klin. Notwendigkeit | |  |  |  |  |
|  | **Plastische Chirurgie und Neurochirurgie** | bei klin. Notwendigkeit | |  |  |  |  |
|  | **Psychiatrische Vorstellung** | Bei Bedarf | |  | Erwägen^4^ |  |  |
|  | **Rehabilitation (Skoliose, Onko-Rehab)** | wiederholt, je nach Bedarf | |  |  |  |  |
|  | **Soziale Arbeit** | bei Bedarf | |  |  |  |  |
|  | **Vorstellung interdisziplinäres Neuro-onkolog. Tumorboard** | bei klin. Notwendigkeit | |  |  |  |  |

**Supplementary Figure 1: German version of the Neurofibromatosis Type 1 Adult Surveillance Form for Austria***1Zeichen für MPNST: schnell wachsende oder schmerzende Knoten, neue neurologische Ausfälle, Veränderung der Konsistenz (z.B. neuer Knoten in weichem Neurofibrom). 2 Zeichen für einen ZNS Tumor: neue fokale Symptomatik, neue, starke kontinuierliche Kopfschmerzen, epileptischer Anfall, neuro-kognitive Veränderungen. 3Hinweis auf GIST:Veränderung in der Verdauung, kontinuierliche Bauchschmerzen, ungewollter Gewichtsverlust. 4Falls vorher keine Anbindung an ein Expertise NF1 Zentrum. 5 Hochrisikopatient für MPNST: 1 Kriterium erfüllt: vorhergegangenes atypisches Neurofibrom (ANNUBP) od. hohe interne Tumorlast bzw. große oder multiple plexiforme Neurofibrome od. st.p. Strahlentherapie od. ein Verwandter mit NF1 und MPNST od. NF1-Mikrodeletion (incl. SUZ12) od. Missense Variante betreffend Codons 844-848. 6Bei komplexen Fällen, unklarer Bildgebung, high risk Konstellation. ANNUBP, atypical neurofibromatous neoplasm of uncertain biological potential; CT, computed tomography; EEG, electroencephalography; EKG, electrocardiography; FDG-PET, fluorodeoxyglucose positron emission tomography; GF, visual field; GIST, gastrointestinal stromal tumor;MPNST, malignant peripheral nerve sheath tumor; MRT, magnetic resonance imaging; NF, neurofibromatosis; OCT, optical coherence tomography; RR, blood pressure; WS, spinal; ZNS, central nervous system*
